# Tremor assessment using smartphone sensor data and fuzzy reasoning

**DOI:** 10.1186/s12859-021-03961-8

**Published:** 2021-04-26

**Authors:** Caro Fuchs, Marco S. Nobile, Guillaume Zamora, Aurélie Degeneffe, Pieter Kubben, Uzay Kaymak

**Affiliations:** 1 Department of Industrial Engineering and Innovation Sciences, Eindhoven University of Technology, Eindhoven, The Netherlands; 2 Erasme University Hospital, Brussels, Belgium; 3 Maastricht University Medical Center, Maastricht, The Netherlands

**Keywords:** Essential tremor, Tremor assessment, Mobile phone sensor data, Computational intelligence, Fuzzy modeling, Fuzzy self-tuning PSO

## Abstract

**Background:**

Tremor severity assessment is an important step for the diagnosis and treatment decision-making of essential tremor (ET) patients. Traditionally, tremor severity is assessed by using questionnaires (e.g., ETRS and QUEST surveys). In this work we assume the possibility of assessing tremor severity using sensor data and computerized analyses. The goal of this work is to assess severity of tremor objectively, to be better able to asses improvement in ET patients due to deep brain stimulation or other treatments.

**Methods:**

We collect tremor data by strapping smartphones to the wrists of ET patients. The resulting raw sensor data is then pre-processed to remove any artifact due to patient’s intentional movement. Finally, this data is exploited to automatically build a transparent, interpretable, and succinct fuzzy model for the severity assessment of ET. For this purpose, we exploit pyFUME, a tool for the data-driven estimation of fuzzy models. It leverages the FST-PSO swarm intelligence meta-heuristic to identify optimal clusters in data, reducing the possibility of a premature convergence in local minima which would result in a sub-optimal model. pyFUME was also combined with GRABS, a novel methodology for the automatic simplification of fuzzy rules.

**Results:**

Our model is able to assess tremor severity of patients suffering from Essential Tremor, notably without the need for subjective questionnaires nor interviews. The fuzzy model improves the mean absolute error (MAE) metric by 78–81% compared to linear models and by 71–74% compared to a model based on decision trees.

**Conclusion:**

This study confirms that tremor data gathered using the smartphones is useful for the constructing of machine learning models that can be used to support the diagnosis and monitoring of patients who suffer from Essential Tremor. The model produced by our methodology is easy to inspect and, notably, characterized by a lower error with respect to approaches based on linear models or decision trees.

## Background

Millions of people worldwide are affected by neurological diseases that induce tremors. Although tremor is often associated with Parkinson’s disease, it can also be a symptom of other neurological disorders. A patient suffering from tremor shows involuntary oscillations of any body part around any plane [1]. When there is no demonstrable cause of this tremor, the patient is diagnosed with Essential Tremor (ET).

Tremor hinders many daily tasks, such as precision tasks like buttoning shirts or other tasks like holding a cup of hot coffee [2]. For the correct diagnosis and treatment of tremor-related diseases, tremor severity assessment is an important step. Tremor severity assessment is also an important tool for evaluating treatment and monitoring how the disease develops, since essential tremor is a chronic and progressive neurological disease. Up to now, tremor severity is measured with qualitative rating scales, such as the Quality of life in Essential Tremor (QUEST) score or the Essential Tremor Rating Scale (ETRS) score.

The QUEST score [3] is determined by making the patient fill in a 30-item questionnaire that contains questions related to the effect of the tremor on the patient’s quality of life. Examples of such questions are “My tremor interferes with my ability to communicate with others” and “I have lost interest in my hobbies because of my tremor”.

The ETRS [4] is filled by a medically trained assessor and evaluates the severity of the tremor in rest, postural, and during movement. The assessor asks the patient to perform 21 different tasks (such a pouring a cup of water, or drawing a line or spiral) and scores the severity of the tremor during these tasks on a 0–4 scale.

Both the QUEST and the ETRS scores are widely used for diagnostics and treatment of ET, but they are subjective in nature. Using direct measurements from wearable sensors could provide more objectivity of the tremor severity assessment. Therefore, the attention for computerized tremor analysis has increased recently (for example, see [5–8]).

These previous studies often rely on dedicated devices to measure the tremor. These devices are expensive and only useful in clinical settings since patients do not have these devices at home. In this study, we propose using the relatively cheap and widely available sensors of smartphones to measure tremor severity. To do so, we rely on the smartphone application TREMOR12 [9], an open-source mobile app was developed by clinicians from the Maastricht University Medical Center. The app is freely available for research purposes in the Apple App Store.

In this study, we aim to develop a fuzzy model that maps the relationship between the QUEST and ETRS score and the sensor measurements of a smartphone strapped to the wrist of an ET patient. We use machine learning to determine the optimal parameters of the model. While many machine learning models (such as deep neural networks) behave like black boxes, fuzzy models have the advantage of being transparent. Because of this property, humans can inspect and study the behavior of the model. The resulting model can help clinicians to more objectively assess the severity of the patient’s tremor and can increase the inter-rater reliability among clinicians.

## Related work

A literature study by Grimaldi and Manto [10] reveals that four different types of sensors are commonly used to measure tremor for in ET patients:

1. *Accelerometers* assess the linear acceleration on a specific axis (in g). These measurements are a combination of linear acceleration, gravity, and additive noise. To diagnose tremor, only the linear acceleration component is relevant, but up to now, no analytic model has been validated to separate gravity (and noise) from the linear acceleration. Low-pass filtering is currently used in most studies [10].
2. *Gyroscopes* measure how fast the angular position or orientation of an object changes over time. Because of this, gyroscopes are used to measure the rotation speed (in radians per second) and the rotation or orientation (in radians).
3. *Elektromyograms (EMGs)* are used to measure the activity of the muscle fibers. This data is collected by a fine wire, which is inserted into a muscle of the ET patient. In [11], researchers were successful in differentiating Parkinson’s disease from Essential Tremor utilizing an Elektromyogram.
4. *Force sensors* assess the torque in newton meter and angular motion (in radians per second). The torque is the force applied to rotate an object around a pivot or axis. Force meters are assumed to be promising for assessing tremor severity, but the measuring devices are non-portable and expensive.

In Table 1 the characteristics of each of the before mentioned sensors for assessing tremor severity are summarized. Both the accelerometer and the gyroscope are affordable and non-invasive, which makes them easy to use on a larger scale. Therefore, studies that utilize these types of sensors will be the focus of the remainder of this section.

**Table 1:** Characteristics of sensors to measure tremor severity

	Accelerometer	Gyroscope	EMG	Force sensors
Gravitational component	Yes	No	No	No
Signal to noise ratio	Low to high	High	High	High
Size of the sensor	Small	Small	Small	Large
Easy to use	Yes	Yes	Variable	No
Invasive	No	No	Yes	No
Cost	Cheap	Cheap	Cheap	Expensive

In a pilot study [8], the feasibility of assessing the severity of the symptoms and motor complications for Parkinson’s disease patients by means of accelerometer data is investigated. Twelve patients were asked to wear a body sensor network that consisted of 8 accelerometers: two sensors were located on the upper arms (one right, one left) two on the lower arms, two on the upper legs, and two sensors were located on the lower legs of each patient. The data that was gathered was used to train a support vector machine which classified the severity of tremor, dyskinesia, and bradykinesia. For validation, the output of this classification model was compared with the assessment of medically trained clinicians. The error of the model (compared to the clinician’s estimates) were on average 2.5%.

In another study [12] on the usefulness of accelerometer data, data of 18 Parkinson’s Disease patients and 5 control patients were gathered. During the data measurement of both resting and action/postural tremor, a set of accelerometers was mounted on different patient’s body segments. The estimation of the tremor type (resting or action/postural) and the severity of the tremor are based on features extracted from the acquired accelerometer signals and hidden Markov models. The trained models were able to quantify tremor severity with 87% accuracy, discriminate resting from postural tremor, and discriminates tremor from other Parkinsonian motor symptoms during daily activities.

A similar study [7] data of 7 Parkinson’s Disease patients were collected using wrist-worn accelerometers and gyroscopes. These sensor data were used to train a least-square-estimation model to estimate the severity of rest, postural, and kinetic tremors. The models’ estimation of tremor severity correlated well with the estimations of a neurologist (r = 0.98).

The above-mentioned studies show great potential for automatic tremor severity assessment, but all studies make use of dedicated devices to measure the tremor severity. These devices are often expensive, hard to use, or not widely available. To overcome this problem, some studies propose to make use of smartphone sensors to measure tremor severity (e.g. [13–15]). However, these studies (and the smartphone applications used) focus on Parkinson’s disease patients. In Table 2 it can be observed that tremor that stems from Parkinson’s Disease differs significantly from Essential Tremor. To our knowledge, only one study [6] used smartphone sensors for gathering data to estimate the severity of Essential Tremor. However, only accelerometer data is used in this study.

In this work, we aim to build a model that is able to estimate tremor severity (in terms of ETRS or QUEST score) from smartphone sensor data. In contrast to [6], we use both the accelerometer and gyroscope of a smartphone. For data gathering, the freely available TREMOR12 application [9] is used.

**Table 2:** Characteristics of tremor caused by Parkinson’s disease and essential tremor

	Parkinson’s disease	Essential tremor
Tremor	Resting	Postural and kinetic
Frequency of tremor	4–6 Hz	7–12 Hz
Presence in hands	>70%	>95%

## Method

### Data collection

In this study we make use of data that are recorded using the the TREMOR12 application [9]. This app offers low-cost tremor quantification for research purposes and algorithm development. With TREMOR12 it is possible to record acceleration, rotation, rotation speed, and gravity, all along three axes. The measurements are all time-stamped, and it is possible to sample in a frequency of up to 100 Hz. The raw signal data of the sensors can be exported as a comma-separated file (*.csv*) for further processing and analysis. The application has been developed by clinicians (from the Maastricht University Medical Center, the Netherlands) to aid in evaluating the effect of the treatment of Essential Tremor patients. TREMOR12 runs on iPhone and iPod Touch (iOS8 or newer) and can be downloaded free of charge from the App Store. The source code is freely available from GitHub at the following address: https://github.com/digneurosurgeon/tremor12.

The sensor data are collected with a 10-ms sample rate (100 Hz). The measuring device is an iPhone 5s. Samples are collected using the accelerometer and the gyroscope of the phone, and measurements are done along the x, y, and z axes. The recorded variables are acceleration (in g) and rotation speed (in radians per second).

**Fig. 1:**
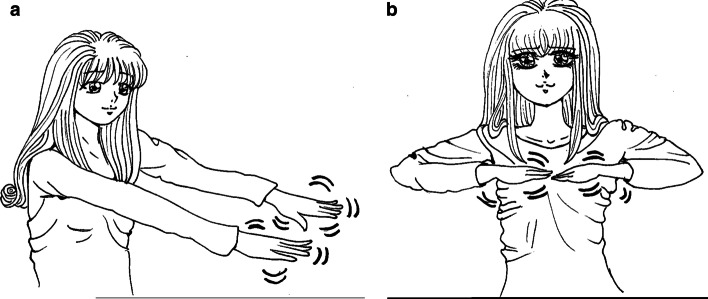
Body positions for data measuring (figure adapted from [16])

Twenty participants (11 men, 9 women), who were all diagnosed with Essential Tremor, took part in the experiment. At the time of the experiment, the average age of the participants was 67 years, and they were on average diagnosed with Essential Tremor 22 years ago. All participants underwent Deep Brain Stimulation as a treatment for Essential Tremor in the past. The ETRS scores of the participants varied between 10 and 68 and the time of the experiment, their QUEST scores were between 3 and 68.1 (Fig. 1).

At least 95% of the patients who suffer from Essential Tremor have a tremor in the upper limbs [17]. Because of this, the sensor data recording device for this study is strapped around the participant’s wrists. The phone was attached using an arm strap that is generally used for running and other sports activities. The phone was strapped on the dorsal side of the wrist by the trained experimenter to ensure a good fit and non-rigid fixation.

The participants were asked to perform five different tests. All tests were performed twice by each participant: once using the left hand and once using the right hand. The following tests were conducted:

1. *Rest* The participant was asked to place both forearms and hands resting on a table. The tremor was measured for one minute on both wrists.
2. *Postural 1* The participant was asked to stretch both arms forward with the hand palms facing down. This position is depicted in Fig. 1a. The tremor was measured for one minute on both wrists.
3. *Postural 2* The participant was asked to hold both hands in front of the chest with the hand palm down and elbows sideways. This position is depicted in Fig. 1b. Again, tremor is measured on both sides for 1 min.
4. *Glass* The participant was asked to pick up a glass of water from the table, bring the glass towards the mouth, and put it back on the table to measure the kinetic tremor. This test was repeated three times for each side.
5. *Finger-nose test* The participant was asked to bring his index finger to the nose and subsequently to the index finger of the researcher. This movement was repeated three times to measure the kinetic tremor.

### Data preparation

#### Filtering out noise

**Fig. 2:**
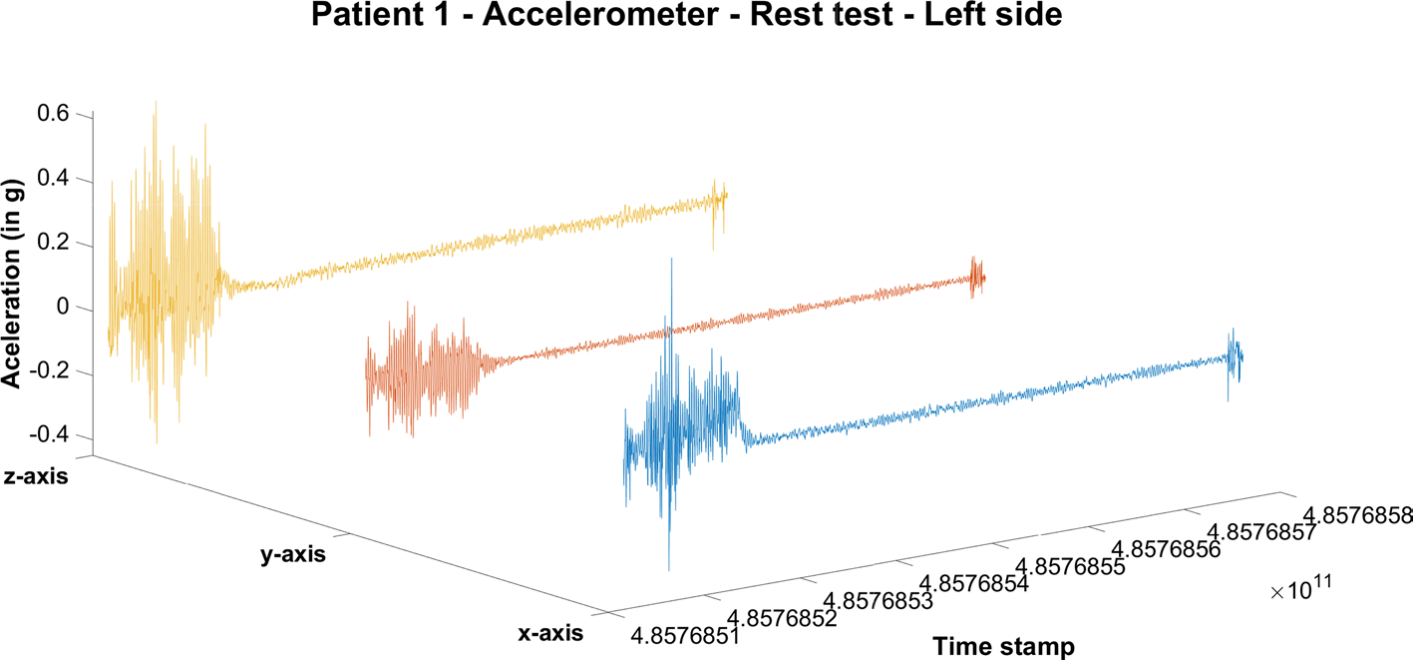
Acceleration for patient 1 during the rest test on the x, y and z axis. Tapping the start and stop recording button on the device and the patient getting in the correct position creates noise in the data

To begin and terminate the recording of data, the user must tap a button on the screen of the recording device. This procedure introduces noise at the beginning and at the end of the recording, as highlighted in Fig. 2.

According to our analyses, this type of noise is typically present in the first and the last 50 samples of the data recorded during each test. Hence, in all tests that follow, the first and last 50 samples of each signal file were automatically discarded.

**Fig. 3:**
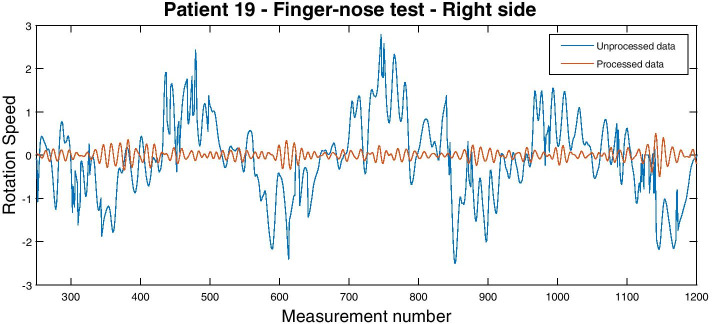
Patient 19—finger-nose test—right hand: The signal before (blue) and after (red) equiripple FIR filtering

An additional source of noise in the measurements is due to voluntary movement, as evidenced during glass and finger-nose test. An example of such noise is shown in Fig. 3, where the original unprocessed signal is denoted by the blue line. Wide, low-frequency peaks originate from the patient moving his finger from his nose to the researcher’s hand and vice versa, a movement that was repeated three times. This signal is not relevant for tremor assessment and must be removed. In order to filter out the noise due to voluntary movement, we applied an Equiripple Finite Impulse Response filter between 7 and 12 hertz, corresponding to Essential Tremor frequencies [1]. In this way, gravitational and motion components are removed from the signals. The result of this filtering phase is denoted in Fig. 3 by a red line.

### Feature extraction

When all signals are processed and filtered, we proceed with the feature extraction phase. The goal is to reduce the time-series to features values, i.e., scalar real values encompassing all the information that can be used in the learning phase. Specifically, the following features are extracted:

- in the time domain, we calculate the root-mean-square (RMS) value of the signal which is a measure for the signal strength, and the signal period, which represents the average duration of one wave in the signal;
- in the frequency domain, the dominant magnitude and frequency are extracted using Daubechies 8 wavelet [18]. The power growth is also extracted from the time-frequency domain to represent the power increase over time.

These features provide information about the highest intensity peak accumulated over the frequency range and the number of oscillations per second associated with it. Overall, 50 features (left and right wrist $\times$ five tests $\times$ five features per signal) must summarize the tremor for every patient.

### Feature selection

Not all variables need to be meaningful when mapping the sensor data to the QUEST or ETRS score. We would like to find the smallest possible subset of variables that still provides a good mapping. To identify the most relevant variables for the models, we adopt a wrapper approach (for more information, see [19, 20]). We employ sequential forward selection in combination with cross-validation, which means variables are sequentially added to an empty candidate set until the addition of new variables does not decrease mean square error (MSE) of the model. The underlying models are CART decision trees [21] since these models are easy and fast to train and have been proven to be effective in modeling using sensor data of Essential Tremor patients [22].

### Missing data

Not all tests have been performed by all patients since the finger-nose test was added to the experiment only after the data of the first four patients were already collected. Spline interpolation was used to estimate the missing values in case of incomplete data series.

### Fuzzy modeling

### FST-PSO clustering and fuzzy set inference

The first stage of our methodology consists of performing a fuzzy clustering [23] on the experimental data, in order to map such clusters against the linguistic variables. This step is necessary to automatically derive the fuzzy sets for the fuzzy rules. The conventional fuzzy C-means algorithm can be prone to premature convergence to local minima [24]. For this reason, we developed a novel version of the clustering algorithm powered by the swarm intelligence method Fuzzy Self-Tuning PSO (FST-PSO) [25]. FST-PSO is a settings-free variant of Particle Swarm Optimization, able to adapt its functioning to the problem under investigation. In this case, FST-PSO contributes to determining the optimal cluster centers and the corresponding fuzzy partition matrix. By projecting the elements of the clusters on the linguistic variables, and by calculating the envelope of membership values for each cluster, fuzzy sets can be created and fit automatically. Specifically, in this work we fit Gaussian fuzzy sets. These fuzzy sets are used later by our system to represent linguistic terms.

### Takagi–Sugeno fuzzy model

Fuzzy models are “expert systems”, where knowledge is represented in the form of (fuzzy) rules [26]. The knowledge is formalized using fuzzy sets [27] and their membership functions, which are exploited in these fuzzy rules. During the process of fuzzy inference, a given input is mapped to the output. This mapping serves as a basis from which decisions or predictions can be made. Because the fuzzy rules are presented in natural language, they can be inspected and interpreted by humans, so that they can reveal patterns in the data.

In this work, we develop a first-order Takagi–Sugeno fuzzy model. A Takagi–Sugeno fuzzy model [28] consists of a set of fuzzy rules. Each rule characterizes a local relation between the input and output variables. The rules of a first order Takagi–Sugeno fuzzy model are all in the following format:

1 $$\begin{aligned} {\mathbf {R}}_j:&\text{ IF } x_{1} \text{ is } A_{j1} \text{ and } \dotsc \text{ and } x_{N} \text{ is } A_{jN} \\&\text{ THEN } y_{j} = {\mathbf {a}}_j^{T}{\mathbf {x}} + b_{j} \end{aligned}$$

In this representation $j = 1, \dotsc J$ denotes the rule number and ${\mathbf {x}} = (x_{1}, \dotsc x_{N})$ is the input vector. *N* is the number of input features; $A_{jn}$ denotes the fuzzy set for rule $R_j$ and $n^{th}$ feature, and $y_j$ is the consequent function of rule $R_j$, that is, a linear combination of the elements of ${\mathbf {x}}$, with coefficients ${\mathbf {a}}_j$ and a constant $b_{j}$.

To assess to which degree each of the fuzzy rules applies in specific cases, the degree of fulfillment is calculated. The degree of fulfillment of a rule *j* is defined as:

2 $$\begin{aligned} \beta _j = \min (\mu _{A_{j1}} ({\mathbf {x}}),\ldots , \mu _{A_{jN}} ({\mathbf {x}})), \ \text {for } j=1,\dots ,N. \end{aligned}$$

The overall output $y^*$ of the fuzzy model for a specific vector of input variables $\mathbf{x }$ can then be calculated as the weighted average of the outputs of the individual rules:

3 $$\begin{aligned} y^*= \frac{\sum _{j=1}^{J} \beta _j y_j}{\sum _{j=1}^{J} \beta _j}. \end{aligned}$$

The creation of a Takagi–Sugeno fuzzy model generally takes place in two steps [29, 30].

- In the *structure identification* phase, the number of rules and partition of the feature space is determined. This is often done by employing clustering algorithms, e.g., grid partitioning, subtractive [31] clustering, or fuzzy c-means [32]. In this study, we apply FST-PSO clustering (as described in the previous section);
- In the step of *parameter identification*, the model’s parameters (e.g., the membership functions and the linear coefficients) are identified. Often least-square or derivative-based optimization techniques are used for this (for more information see [29]). In this work we rely on least-square optimization techniques.

### Simplification of the rule base with GRABS

Fuzzy inference systems (FIS) found application in several fields of science over the last years because of their transparency and interpretability, which is higher than common “black-box” machine learning approaches. In order to estimate a transparent FIS from data, we proposed a new approach, named GRABS (Graph-Based Simplification), for FIS simplification which leverages graph theory to identify and remove similar fuzzy sets from rule bases [33]. GRABS uses a threshold-based similarity index (the Jaccard similarity [34]) to determine which fuzzy sets are overlapping. Then, a graph is created, where the fuzzy sets are the nodes, and similar sets are linked with an edge. GRABS detects the connected sub-components in the graph, whose nodes represent the same linguistic term. It then proceeds to the simplification (i.e., removal) of redundant nodes. Thanks to the similarity threshold hyper-parameter, GRABS can be used to reduce the bloating of rules with a tunable level of error.

### Software

For this work, we relied on two Python packages: Simpful and pyFUME. In this section, we give some background on the workings of these packages.

#### Simpful

Simpful is a user-friendly, general-purpose, lightweight Python API for the definition of FIS [35]. Simpful’s API was designed in such a way that the modeling phase should resemble, as much as possible, human thinking and natural language, in order to simplify the definition of fuzzy sets, linguistic variables, and fuzzy rules. Simpful also supports fuzzy inference based on Sugeno reasoning of any order. One key feature of Simpful is that fuzzy rules are defined through well-formed strings of text, expressed using natural language, and thus simplifying the definition of the rule base. Thanks to the human-readable format, Simpful facilitates the inspection of the model and the interpretation of results.

The current version of Simpful (v2.0.0) supports both *(i)* polygonal and functional (e.g., sigmoidal, gaussian or custom shaped) fuzzy sets. Simpful also supports the definition of fuzzy rules with an arbitrary degree of complexity, built using linguistic terms combined with logic operators (e.g., AND, OR, NOT). Simpful supports the definition of FIS with multiple inputs and outputs, with no limitation, and the definition of arbitrary fuzzy networks [36]. pyFUME, described in the next paragraph, creates Simpful models automatically out of data. Simpful is available for download, under GPL license, on GitHub at the following URL: https://github.com/aresio/simpful. Simpful can also be installed by using the PyPI facility: pip install simpful.

#### pyFUME

pyFUME [37] is a Python library for the automatic derivation of fuzzy models, designed to provide an easy to use and extensible interface both for practitioners and researchers.

Currently, pyFUME offers facilities to simplify the following operations: loading of the input data, with automatic partitioning between training and test data sets; clustering of the data in the input-output space by means of Fuzzy C-Means (FCM) clustering [32] or the method based on Fuzzy Self-Tuning Particle Swarm Optimization (FST-PSO [24, 25]) described in the previous sections; estimation of the antecedent sets of the fuzzy model, using the method described in [38], using Gaussian (default option), double Gaussian, or sigmoidal membership functions; estimation of the consequent parameters of the first-order TS fuzzy model, implementing the functionalities described in [39]; generation, using the estimated antecedents and consequents, of an executable Simpful model (with the possibility of exporting the source code as a separate, executable file). pyFUME also provides a facility for the testing of the derived model, providing functionalities for the measurement of Root Mean Square Error (RMSE), Mean Square Error (MSE), or Mean Absolute Error (MAE).

pyFUME is implemented in Python, depends on numpy [40], scipy [41] and Simpful [35], and can be downloaded from GITHUB at the following address: https://github.com/CaroFuchs/pyFUME. pyFUME can also be installed by using the PyPI facility: pip install pyFUME.

## Results

**Fig. 4:**
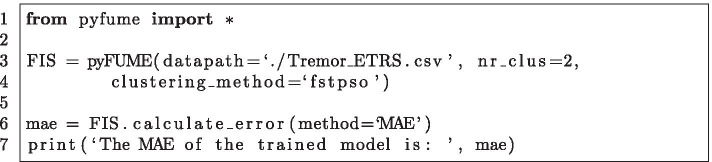
The pyFUME code to generate the model that maps the sensor data to the ETRS score (line 3–4) and to calculate the mean square error of the resulting model (line 6–7)

We generated two fuzzy models using pyFUME: one to map the sensor data to the ETRS score and one to the QUEST score. The data set at hand for this study is limited in size. Because of this, we limit both models to two rules to avoid overfitting. For structure identification, FST-PSO based clustering was used [24]. The model was simplified using GRABS, with a similarity threshold of 0.90, since this number simplifies the models sufficiently without losing information and compromising on accuracy. All other settings for pyFUME were kept on default. A summary of these settings can be found in Table 3. The pyFUME code to generate the model and calculate the mean absolute error can be found in Fig. 4.

**Table 3:** Parameter settings used for developing the fuzzy models

*Data preparation*	
Data normalization	Yes
Percentage used for training/testing the models	75%/25%
*Model structure*	
Number of clusters	2
Shape membership functions	Gaussian
T-norm	Minimum
*Model construction*	
Clustering method	FCM coupled with FST-PSO
Degree of fuzziness of the identified cluster structure (*m*)	2
FST-PSO maximum number of iterations	100
GRABS threshold [33]	0.9

### ETRS score

In our previous work [22] we show that following features are related to the ETRS score (see also Table 4 for an overview): the power growth of the accelerometer data in rest, the dominant magnitude of the accelerometer and the signal root mean square (RMS) of the rotation speed as measured during the first postural test, and the dominant frequency (of the accelerometer) and the dominant magnitude (of the rotation speed) as measured during the second postural test. Therefore, these five variables are used to generate the fuzzy model that maps the sensor data to the ETRS score. The following two rules were obtained:

- RULE 1: IF (dominantFrequency IS high) AND (dominantMagnitude IS any value) AND (signalPeriod IS any value) AND (powerGrowth IS any value) THEN (ETRS score = 60.40 * dominantFrequency − 87.00 * dominantMagnitude + 14.0 * signalPeriod + 7.10 * signalRMS + 57.70 * powerGrowth - 2.19)
- RULE 2: IF (dominantFrequency IS low) AND (dominantMagnitude IS low) AND (signalPeriod IS medium) AND (powerGrowth IS medium) THEN (ETRS score = − 33.30 * dominantFrequency + 41.30 * dominantMagnitude + 10.80 * signalPeriod + 3.19 * signalRMS + 8.53 * powerGrowth + 55.70)

**Fig. 5:**
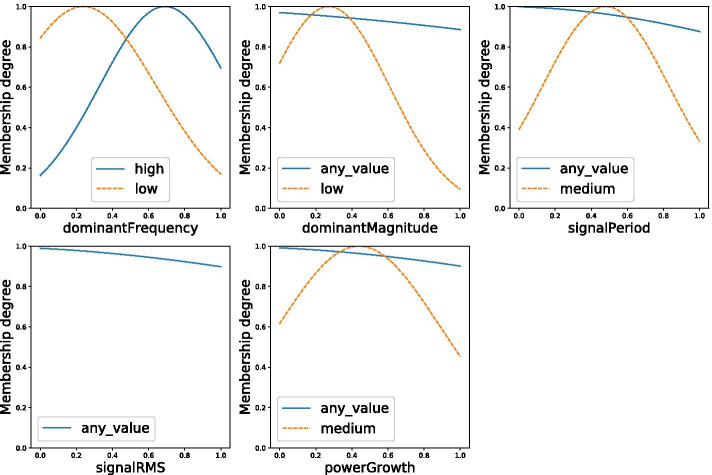
The membership functions for the model that maps the sensor data to the ETRS score

This model maps the sensor data to the ETRS score with a mean absolute error (MAE) of 2.70. The membership functions belonging to this fuzzy model are plotted in Fig. 5. As can be observed in the model’s rules and Fig. 5, one of the two fuzzy sets for the variable ‘signal RMS’ has been dropped by GRABS. This means the two sets showed a similarity of more than 90%.

Figure 5 seems to imply that the variable ‘Signal RMS’ does not contribute to the the performance of the system. To test this hypothesis, a new model excluding this variable is trained. The resulting rules are as follows:

- RULE 1: IF (dominantFrequency IS high) AND (dominantMagnitude IS any value) AND (signalPeriod IS any value) AND (powerGrowth IS any value) THEN (ETRS score = 27.8 * dominantFrequency − 42.4 * dominantMagnitude + 22.4 * signalPeriod + 23.3 * powerGrowth + 8.7)
- RULE 2: IF (dominantFrequency IS low) AND (dominantMagnitude IS low) AND (signalPeriod IS medium) AND (powerGrowth IS medium) THEN (ETRS Score = − 10.8 * dominantFrequency + 53.5 * dominantMagnitude − 18.2 * signalPeriod + 14.0 * powerGrowth + 58.5)

**Fig. 6:**
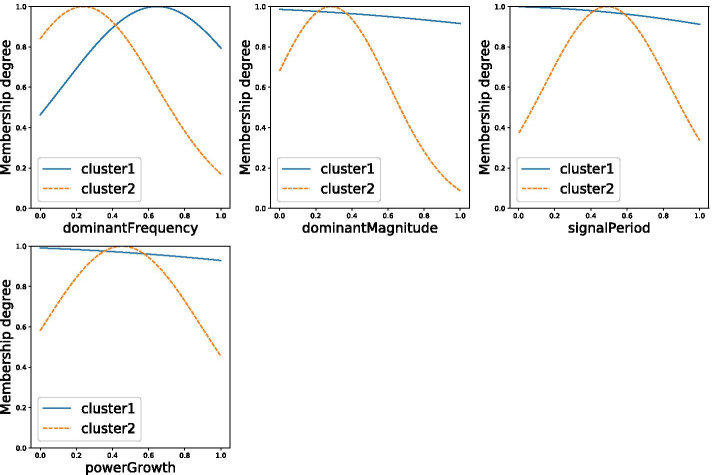
The membership functions for the model that maps the sensor data to the ETRS score, excluding the variable ‘Signal RMS’

The membership functions belonging to this fuzzy model are plotted in Fig. 6. This model has a mean absolute error (MAE) of 1.85. It can therefore be concluded that this model outperforms the previously presented model.

The model excluding ‘Signal RMS’ performs better than a multiple regression line ($R^2 = 0.58$, MAE = 8.44). We can also conclude that this model outperforms the model presented in [22], which was trained on the same data set and had an MAE of 6.41. Therefore, compared to the model presented in [22], the error has been reduced by 71.1%. These results are also shown in Table 5.

The computational time required to estimate the ETRS for new patients using these models is negligible . For example, computing the output of the fuzzy model—which is the most complex model of the three models that were developed—requires 0.6 ms (tested on a machine running Windows 10, with a Intel® Core™ i7-7700HQ CPU @ 2.80GHz).

### QUEST score

Following the findings presented in [22], we select the power growth of the rotation speed during the first postural test and the dominant frequency of the accelerometer as measured during the second postural test (see Table 4). These variables are then used to train the fuzzy model that maps the sensor data to the QUEST score. The trained model consist of the following set of rules:

- RULE 1: IF (DominantFrequency IS high) AND (PowerGrowth IS any value) THEN (QUEST score = 4.66 * DominantFrequency − 8.52 * PowerGrowth + 11.70)
- RULE 2: IF (DominantFrequency IS low) AND (PowerGrowth IS low) THEN (QUEST score = − 4.41 * DominantFrequency − 69.50 * PowerGrowth + 47.00)

**Fig. 7:**
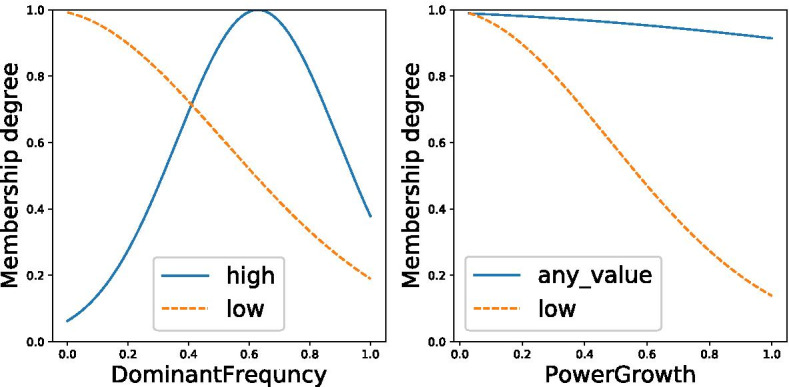
The membership functions for the model that maps the sensor data to the QUEST score

The membership functions of this fuzzy model are plotted in Fig. 7. None of the fuzzy sets showed high similarity, and therefore none of the sets were dropped by GRABS.

The mean absolute error of the fuzzy model is 2.30. When using the same data to fit a multiple regression line, a low fit is found ($R^2 = 0.17$), which also leads to low performance (MAE = 12.10). The fuzzy model therefore clearly outperforms the regression line. The decision tree as presented in [22] has an error of 8.65. This means an improvement of 73.4% in terms of the error rate for the fuzzy model compared to a decision tree when using the same data. These results are summarized in Table 5.

The computational time required to estimate the QUEST score for new patients using the fuzzy model is 0.4 ms (tested on a machine running Windows 10, with a Intel® Core™ i7-7700HQ CPU @ 2.80GHz).

**Table 4:** Variables used for training the fuzzy models

ETRS			QUEST		
Test	Sensor	Feature	Test	Sensor	Feature
Rest	Accelerometer	Power growth	Postural 1	Rotation speed	Dominant frequency
Postural 1	Accelerometer	Dominant magnitude	Postural 2	Accelerometer	Power growth
Postural 1	Rotation speed	Signal RMS			
Postural 2	Accelerometer	Dominant frequency			
Postural 2	Rotation speed	Dominant magnitude

**Table 5:** Comparison between the regression models, decision trees and fuzzy models for predicting the ETRS and QUEST score. The fuzzy models outperform the other two models

	ETRS			QUEST		
	Regression line	Decision tree [22]	Fuzzy model	Regression line	Decision tree [22]	Fuzzy model
MAE	8.44	6.41	**1**.**85**	12.10	8.65	** 2.30**

Best values in bold

## Discussion

We presented a data-driven methodology for the automatic creation of interpretable models supporting the diagnosis and monitoring of patients suffering from Essential Tremor. Our methodology relies on pyFUME [37]—a novel tool combining swarm intelligence, fuzzy clustering, and fuzzy reasoning—extended with GRABS, a graph-based approach for rule-base simplification [33].

The tremor data used in this study was collected using the TREMOR12 application, which runs on widely available devices (in this study, iPhones), a characteristic that makes it easy and relatively inexpensive to acquire experimental data.

The developed fuzzy models show a better performance than linear models or the CART decision trees presented in [22]. The mean absolute error of the fuzzy model that maps sensor data to the ETRS score is 78% lower compared to the error of a linear model, and 71% lower then decision trees. The fuzzy models also outperform linear models (with 81%) and decision trees with (with 73%) when mapping the sensor data to the QUEST score. The fuzzy models therefore clearly outperform these other models.

In this work we relied on the feature selection phase performed by the CART algorithm in our previous study [22]. However, we envision that this step should be performed autonomously by pyFUME, either as a pre-processing phase or using a bottom-up approach. Integrating this functionality in pyFUME will be among our prioritary future developments.

All the patients involved in this study were treated with DBS during sample acquisition, leading to relatively low values for both ETRS and QUEST scores. Hence, patients suffering from severe tremors were not represented in our data set. In order to make our methodology able to detect and diagnose such cases, additional data collected from untreated patients should be added to the data set, and puFUME should run again to build a model suitable for the identification of such patients.

## Conclusion

The QUEST and the ETRS scores are widely used for diagnostics and treatment of ET, but they are subjective in nature. To overcome this problem, in this study we use direct measurements from wearable (smartphone) sensors to assess the severity of the tremor more objectively. We used the data extracted from these measurements to develop fuzzy models that map the relationship between the QUEST and ETRS score. The models developed in this study outperform both linear models and decision trees, and the running time of model inference is in the order of miliseconds.

Models such as the ones developed in this study can help clinicians to quickly and objectively measure the effect of Deep Brain Simulation (DBS) on the patient’s tremors. For DBS, a medical device called a neurostimulator is implanted in the patient’s chest. The neurostimulator sends electrical impulses trough electrodes to specific brain nuclei, which suppresses the patient’s tremor. The immediate feedback of sensor data and machine learning models could help finding the optimal voltage and the sequence of the stimulation for a specific patient to minimize the patient’s tremors. This could ultimately lead to a situation where the neurostimulator is self-learning, and the settings of the stimulation can be adjusted automatically based on real-time feedback.

The use of sensor data from wearables, combined with models produced by machine learning algorithms, can also be useful for patients treated in different ways. For example, they can be used to find the correct dosage for ET patients treated with medication or measure the effectiveness of magnetic resonance (MR) guided focused ultrasound.

As a final remark, our data set currently contains 20 ET patients: we plan to collect new samples in the future to derive more robust models. This current study shows that using smartphone data can be useful to diagnose and assess the severity of Essential Tremor. The developed models indicate that there is a relation between the smartphone measurements and tremor severity. However, more data is needed to develop models that are suitable for implementation in clinical practice. In the future, we plan to use smaller and lighter devices for data collection in future studies. The size of the measuring device might restrict the movement of the wrist of the patient, which could influence the measurements of the tremor. As a solution to this, we plan to use smartwatches in follow-up studies.

## Data Availability

The data sets used for this study are available from the corresponding author on reasonable request.

## Abbreviations

CART: Classification and regression tree
DBS: Deep brain simulation
ET: Essential tremor
ETRS: Essential Tremor Rating Scale
FST-PSO: Fuzzy self-tuning particle swarm optimization
GRABS: Graph-based simplification
MAE: Mean absolute error
MSE: Mean square error
QUEST: Quality of life in essential tremor
RMS value: Root-mean-squared value
RMSE: Root mean square error
